# Whole-genome sequencing of rough *Brucella melitensis* in China provides insights into its genetic features

**DOI:** 10.1080/22221751.2020.1824549

**Published:** 2020-10-03

**Authors:** Xiaowen Yang, Dongri Piao, Lingling Mao, Bo Pang, Hongyan Zhao, Guozhong Tian, Hai Jiang, Biao Kan

**Affiliations:** a State Key Laboratory for Infectious Disease Prevention and Control, National Institute for Communicable Disease Control and Prevention, Chinese Center for Disease Control and Prevention, Beijing, People’s Republic of China; b Liaoning Province Center for Disease Control and Prevention, Shenyang, People’s Republic of China

**Keywords:** *Brucella*, LPS, whole genome sequencing, phylogenetic analysis, rifampicin resistance

## Abstract

*Brucella* spp. can cause the zoonosis brucellosis, which affects public health and safety and even economic development. *B. melitensis* has a smooth phenotype, while 28 *B. melitensis* isolates had a rough phenotype in 2018. In this study, rough phenotype detection and whole genome sequencing methods were used to analyze the genetic features of rough *B. melitensis*. A drug susceptibility test was also performed. The results showed that the rough *B. melitensis* strains originated from strains isolated in China rather than from foreign strains. Furthermore, an MS tree showed that 9 complexes to be epidemic in China. For the rough *B. melitensis* strains, expression of the metabolic function genes varied in the earlier stages of evolution compared to the cellular process and signalling function genes. Expression of some transcriptional regulatory factors also varied in the later stages of evolution, and compared to MFS transporter genes, ABC transporter genes varied in the earlier stages. Moreover, as there was no significant difference in rifampicin, doxycycline and streptomycin susceptibility between the smooth and rough *B. melitensis* strains, treatment of brucellosis was not affected by strain type. This study provided important information for understanding the genetics and evolution of rough *B. melitensis* in China.

## 1. Introduction

*Brucella* spp. are facultative intracellular pathogens that can persistently colonize animal host cells and cause the zoonosis brucellosis. The main symptoms of brucellosis are fever, sweating, weakness, and joint pain [1]. Severe symptoms cause incapacitated. Brucellosis affects public health and safety and even economic development. Based on biochemical characteristics and host preferences, twelve different species have been identified [2–6]. Most diagnosed human brucellosis cases to date have been caused by *B. melitensis*, *B. abortus*, *B. canis* or *B. suis* [7]. The number of human brucellosis cases worldwide exceeds 500,000 per year, and the incidence of human brucellosis in some endemic countries exceeds 100 cases per million population [8]. However, according to the World Health Organization (WHO), the actual incidence is more than 10–25 times that reported [9].

China has 14 terrestrial bordering countries, most of which have serious cases of brucellosis (https://www.oie.int/wahis_2/public/wahid.php/Countryinformation/Zoonoses). Accordingly, the prevention and control of brucellosis in China is very difficult. The first human brucellosis case in China was reported in Inner Mongolia [10], and brucellosis in humans has peaked twice, in 1957–1963 and 1969–1971. Although brucellosis cases in humans and animals declined significantly during the 1980s to 1990s due to the use of vaccines among animals [11], since the mid-1990s, human brucellosis once again became a threat in China and spread throughout the country [12]. Currently, cases have been reported in all provinces and autonomous regions in China [13], the number of human brucellosis cases in 2019 was 45,406, which was slightly more than that in 2018 (39,296 cases). In the past, the epidemic strains of *Brucella* were *B. melitensis* biovars 1 and 3 [14], though the main isolates in recent years are *B. melitensis* biovar 3 [15].

Except for *B. canis* and *B. ovis*, most *Brucella* spp. have a smooth phenotype under natural conditions. The phenotype of *Brucella* spp. is mainly determined by lipopolysaccharide (LPS) located on the outside of the outer membrane. The LPS of *Brucella* consists of lipid A, core oligosaccharide, and O-antigen, and *Brucella* spp. can be classified into smooth and rough according to whether they contain the O-antigen. The LPS of rough *Brucella* spp. lacks the O-antigen (R-LPS) [16]. Rough and smooth *Brucella* spp. exhibit differences in various aspects, including colony and cell morphology, immunological and biochemical reactions, and virulence, among others. Indeed, rough *Brucella* spp. are more likely to be targets of the immune system due to a lack of protection of lipid A [17], which seriously affects the intracellular survival ability of rough *Brucella* spp. in vivo. These factors led to a reduction in the virulence of rough *Brucella* spp., which do not usually cause brucellosis in humans [18,19].

Many *Brucella* strains isolated from human brucellosis cases are stored by the National Institute for Communicable Disease Control and Prevention of the Chinese Center for Disease Control and Prevention. Among more than 200 strains isolated in 2018, 28 *B. melitensis* isolates had a rough phenotype. Most of them were isolated in Liaoning Province. The rough *B. melitensis* strains in Liaoning Province were mainly isolated from Jingzhou, Huludao and Chaoyang. These three cities were all located in the west of Liaoning Province and closed to Inner Mongolia. Importantly, in Liaoning, the number of brucellosis patients increased in 2018 compared with 2017. These clinical isolates attracted our attention. Therefore, this study was based on all (suspected) rough *B. melitensis* strains and other isolates stored by the Chinese Center for Disease Control and Prevention; whole genome sequencing was used to (1) trace the rough *B. melitensis* strains, (2) determine the genetic characteristics of rough *B. melitensis* strains and (3) assess the sensitivity of rough *B. melitensis* strains to rifampicin, doxycycline, and streptomycin.

## 2. Materials and methods

### 2.1. The strains and genomes of *Brucella* spp. used in this study

The genome, amino acid, and nucleic acid sequences of the public *Brucella* genomes were downloaded from NCBI GenBank (www.ncbi.nlm.nih.gov/genome/?term=brucella) (Table S1). Cultured and inactivated *Brucella* isolations were carried out in a BSL-3 laboratory of the National Institute for Communicable Disease Control and Prevention of the Chinese Center for Disease Control and Prevention. A total of 199 strains isolated in China and 107 foreign strains (including standard strains and *B. melitensis* strains from around the world) were selected.

### 2.2. Rough phenotype detection

First, a slide agglutination test for anti-R monospecific sera was used. R monospecific sera (20 μL) (Department of Inspection Technology Research, China Institute of Veterinary Drug Control, China) were added to clean slides, and an equal amount of a *Brucella* suspension prepared with PBS was added and mixed. The results were observed within 1–2 min. If agglutination occurred, the isolate was a rough *Brucella* spp.; otherwise it was a smooth *Brucella* spp.

Second, the acriflavine agglutination test [20] was also conducted. The method was similar to the method described above, and at least 20 μL of acriflavine was applied. The smooth strains remained in suspension, whereas the rough strains immediately agglutinated. Agglutination was visible in the clear liquid.

Third, phenotypic characterization of the isolated strain was also confirmed by crystal violet staining. The isolated strain was streaked on a plate, which was incubated at 37°C for 5 days and then examined using obliquely reflected light (Henry’s method) before and after staining with crystal violet (White & Wilson’s staining method) [20]. Smooth *Brucella* spp. could not be stained with crystal violet. Positive and negative controls were established for each test.

Finally, the Lipopolysaccharide (LPS) was detected by SDS-PAGE. Bacterial LPS was extracted using the LPS Extraction Kit (iTron, Korea) according to the manufacturer’s instructions. The extracted LPS resuspended in phosphate-buffered saline (PBS) and then heated at 100°C for 2 min. Then the LPS was loaded into a 12% gel for SDS-PAGE test. The LPS of *B. melitensis* 16M and *B. canis* RM6/66 was used as positive and negative controls, respectively.

### 2.3. Whole genome sequencing

Bacterial genomic DNA was extracted using the Wizard Genomic DNA Purification kit (Promega, USA) according to the manufacturer’s instructions. Library preparation was performed using the Nextera XT Library Prep kit (Illumina, USA) according to the manufacturer’s manual. The libraries were sequenced using an Illumina/Solexa sequencing analyzer to 100-fold (100×) genome coverage at the Beijing Genomics Institute (BGI) (Shenzhen, China).

### 2.4. SNPs identification and annotation

SNPs and indels identification and annotation were performed as described in a previous study [21], with minor modifications. We removed SNPs with a quality score of less than 1000. Some SNPs (not all) was identified by PCR amplification and sequencing.

### 2.5. Clustering analyses and phylogenetic tree construction

One genome that had the highest coverage for all isolates was selected as the reference. PhyloSNP [22] was used to generate phylogenetic trees. The neighbour-joining (NJ) method with 1000 bootstraps was used.

SNP matrix data were also analyzed using phyloSNP. A minimum spanning (MS) tree was generated with BioNumerics software (http://www.applied-maths.com/bionumerics) using default software settings. The links between the MS tree nodes represented the distance between the genotypes. The cluster cutoff value was defined as the maximum pairwise distance found between epidemiologically linked isolates.

### 2.6. Rifampicin, doxycycline and streptomycin susceptibility testing

Rifampicin, doxycycline and streptomycin are drugs for brucellosis according to recommendations by the WHO [23]. The susceptibility of a *Brucella* strain to rifampicin, doxycycline and streptomycin (National Institutes for Food and Drug Control, China) was tested by the double dilution method according to CLSI guidelines. Rifampin was double diluted from 0.03–64 μg/mL in 96-well plates. Strains were suspended in saline water to a 0.5 McF turbidity and suspended in *Brucella* broth adjusted to pH 7.1 ± 0.1 (BD, USA). The plate was cultured at 35 ± 2 °C with 5% CO_2_ for 48 h. The quality control strain was *Streptococcus pneumoniae* ATCC 49619 (refer to CISL_M45 (2016)).

### 2.7. Statistical analysis

Statistical analyses were performed using Excel and SPSS. A *P* value < 0.05 was considered significant when using the one-way analysis of variance (ANOVA). The heatmap and other figures were drawn by R software.

## 3. Results

### 3.1. Rough *B. melitensis* originated from Chinese isolates rather than foreign strains

Among 199 China isolates, 68 strains had the anti-R monospecific sera and acriflavine agglutination; 67 strains could be stained with crystal violet; and 64 strains were different from standard strains in SDS-PAGE detection. In summary, there were 64 rough *Brucella* strains were observed in this study according to the results of the rough phenotype detection in this study.

**Figure 1.**
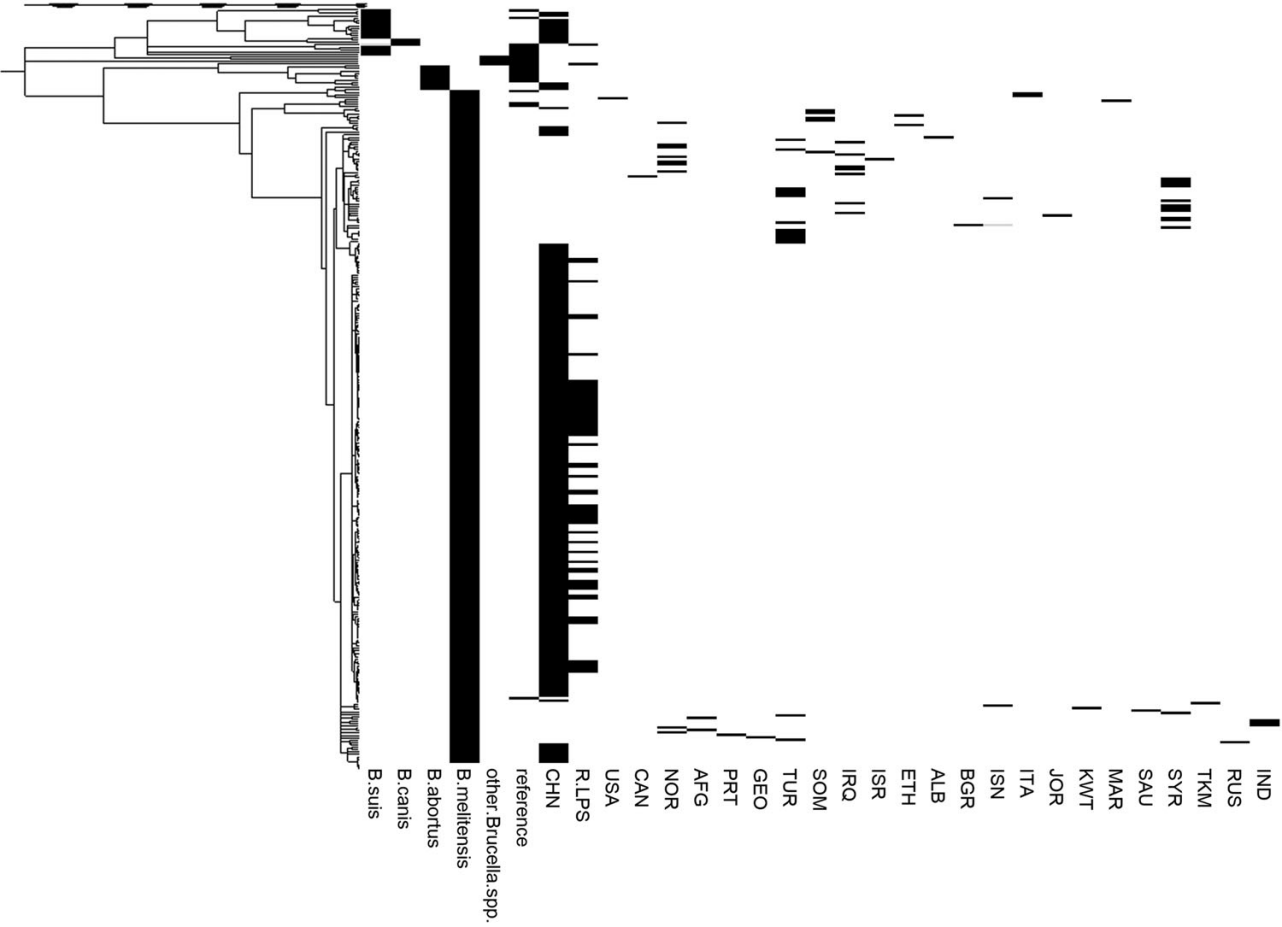
Phylogenetic tree generated by the neighbour-joining method. The phylogenetic tree included the species *B. suis*, *B. canis*, *B. abortus*, *B. melitensis* and other *Brucella* spp. and the locations CHN, USA, among others. R-LPS refers to rough *Brucella* spp., including *B. canis*, *B. ovis* and rough *B. melitensis*. The abbreviations for the countries are shown in Table S1. A total of 306 strains (including 199 strains isolated in China and 107 foreign strains) were used to construct the phylogenetic tree.

The SNPs of whole genomes from 199 strains isolated in China and 107 foreign strains (including standard strains and *B. melitensis* strains from around the world) were selected to construct a phylogenetic tree by the neighbour-joining (NJ) method. As shown in Figure 1, most of the *B. melitensis* strains isolated in China (184 strains) show the highest similarity at the genomic level to *B. melitensis* bv.2 str ATCC 23457. These strains are also highly similar to isolates from the Middle East, but they still grouped into separate clades after refinement. Moreover, 10 strains of *B. suis* were found to mainly belonged to *B. suis* bv.3; 3 strains belonged to *B. abortus*; and 2 strains belonged to *B. canis*. The presence of the rough phenotype was detected, and 68 strains had rough phenotypes. Combined with the NJ tree, a total of 62 rough *B. melitensis* strains were observed in this study (There were 2 strains belonged to *B. canis*). The NJ tree also showed that except for one strain related to *B. melitensis* bv.1 str 16M, the remaining 183 *B. melitensis* strains are similar to *B. melitensis* bv.2 str ATCC23457. Because some smooth and rough *B. melitensis* strains isolated in China grouped into the same clades, strains closely related to the rough *B. melitensis* strains could not be distinguished based on the NJ tree (Figure 1).

**Figure 2.**
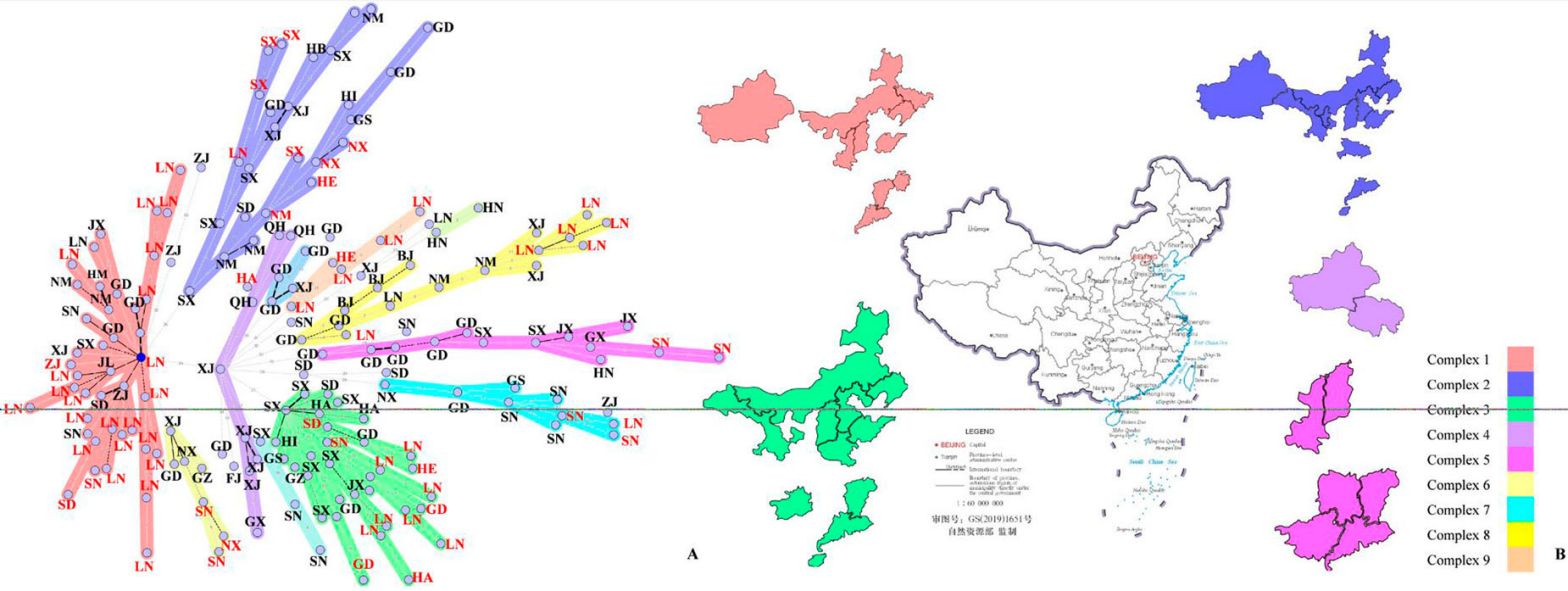
The MS tree and distribution of complexes 1–5. A is the MS tree constructed by SNPs of 183 *B. melitensis* from China. The letters represent the provinces the strains were isolated from which the strains were isolated; red colour indicates the rough *B. melitensis* strains, and black colour indicates smooth *B. melitensis* strains. BJ, Beijing; FJ, Fujian; GD, Guangdong; GS, Gansu; GX, Guangxi; GZ, Guizhou; HA, Henan; HB, Hubei; HE, Hebei; HI, Hainan; HN, Hunan; JL, Jilin; JX, Jiangxi; LN, Liaoning; NM, Inner Mongolia; NX, Ningxia; QH, Qinghai; SD, Shandong; SN, Shaanxi; SX, Shanxi; XJ, Xingjiang; ZJ, Zhejiang. B shows the distribution of complexes 1–5. The different colours represent different complexes.

A total of 183 strains similar to *B. melitensis* bv.2 str ATCC23457 were chosen for further analysis. GATK was used to select SNPs with a score of more than 1,000 to develop a matrix, and then the matrix was used for MS tree construction. The maximum neighbour distance was 20 (Figure 2A). The results showed that the main epidemic strains of *B. melitensis* in China belonged to 9 complexes, with one isolate from Xinjiang being associated with all complexes.

The strains in complex 1 were found in the endemic areas of brucellosis, and the isolates were the most widespread in China. Complex 1 also contained the largest number of rough *B. melitensis* strains in this study. Among the 43 strains in complex 1, 21 strains are rough *B. melitensis*. Most of the rough *B. melitensis* strains were isolated in Liaoning; these strains were also mainly epidemic rough *B. melitensis* strains isolated in Liaoning. The origins are Inner Mongolia, Xinjiang, Liaoning, Shaanxi, and Shandong, and the strains also existed in Guangdong (Figure 2B).

Among the 32 strains of complex 2, 7 strains are rough *B. melitensis*. The strains in complex 2 are associated with strains isolated in Shanxi. The strains in complex 2 were isolated in mid-west and west of endemic areas of brucellosis in China, especially in Shanxi, Ningxia, Inner Mongolia and Xinjiang. This study also found rough *B. melitensis* strains to be widespread in Ningxia, Shanxi, Hebei and Inner Mongolia. In total, 30 strains in complex 3, with 12 rough *B. melitensis* strains. The endemic areas and trends of complex 3 were found to be similar to those of complex 1 and complex 2, but the epidemic strains isolated in Inner Mongolia concentrated more in complex 3. Other rough *B. melitensis* strains isolated in Liaoning were also grouped into complex 3 (Figure 2B).

Complex 4 and 5, with 11 and 13 strains, respectively, are more similar to foreign strains. The strains in complexes 4 were mainly isolated in Qinghai and Xinjiang. These areas have developed animal husbandry and border Kazakhstan, Kyrgyzstan and other countries with endemic brucellosis. No rough *B. melitensis* strains were found in complex 4. According to the NJ tree (Figure 1), the strains in complex 4 are similar to isolates from Russia and Turkey (minor). The strains in complex 5 tended to be endemic in central and southern China. Two rough *B. melitensis* strains were isolated in Shaanxi. Compared to others, the strains in complex 5 are more closely related to the strains isolated in the Middle East, including Turkey (major), Syria, Iraq, and Iran (Figure 2B).

### 3.2. The rough *B. melitensis* strains are highly associated

**Figure 3.**
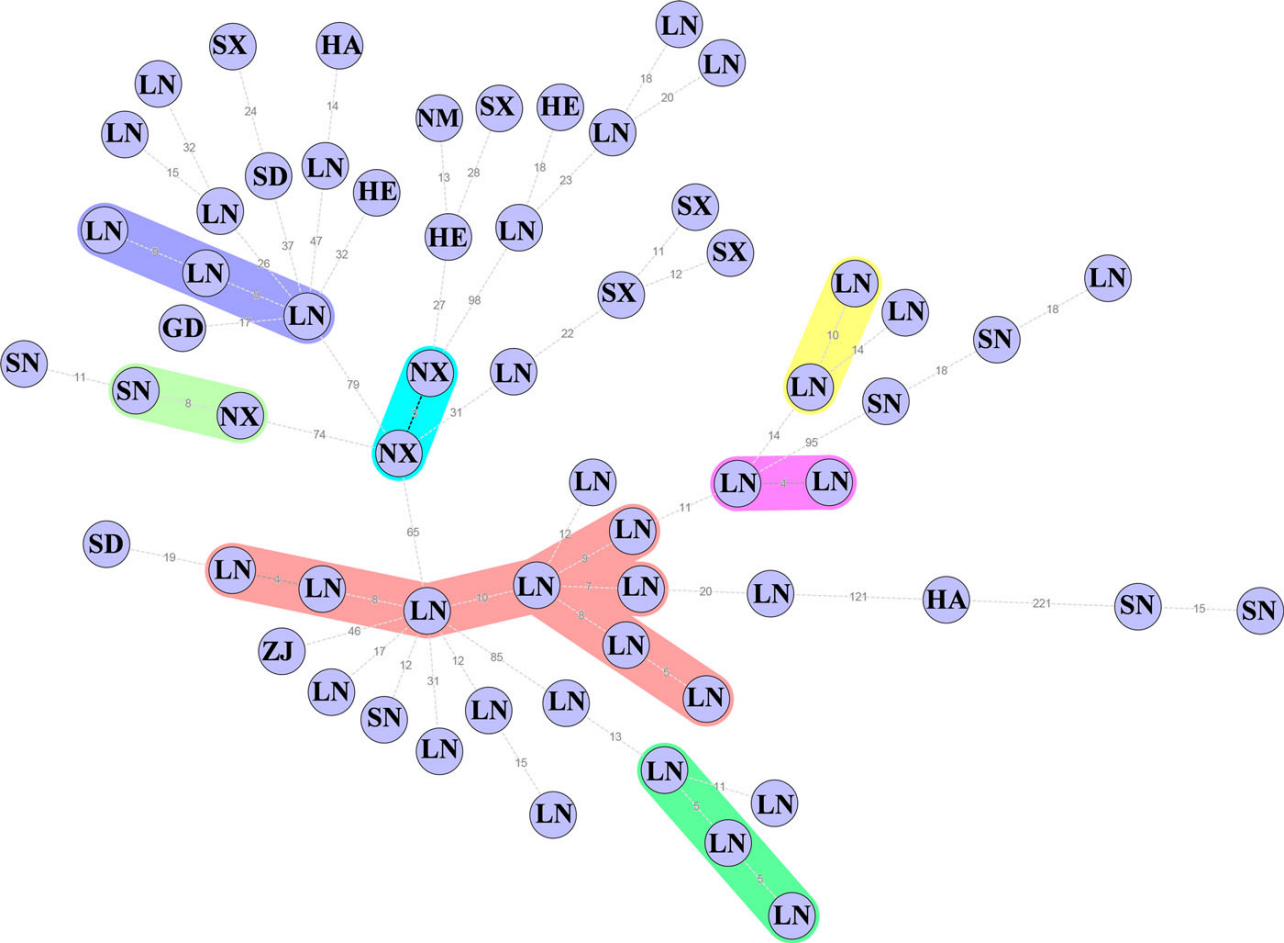
The MS tree of rough *B. melitensis*. The letters represent the provinces the strains were isolated from. GD, Guangdong; HA, Henan; HE, Hebei; LN, Liaoning; NM, Inner Mongolia; NX, Ningxia; SD, Shandong; SN, Shaanxi; SX, Shanxi; ZJ, Zhejiang.

It can be seen from the MS tree that the strain with the highest relationship may be a smooth or rough *B. melitensis* strain. Therefore, the associations of 62 rough *B. melitensis* strains were analyzed separately in this study. The MS tree was also constructed by SNPs, and the maximum neighbour distance was 10 (Figure 3). Compared with isolates from other provinces, the rough *B. melitensis* isolated from Liaoning were more similar. The results showed that the association of rough *B. melitensis* was high, with an epidemic trend in Liaoning.

**Figure 4.**
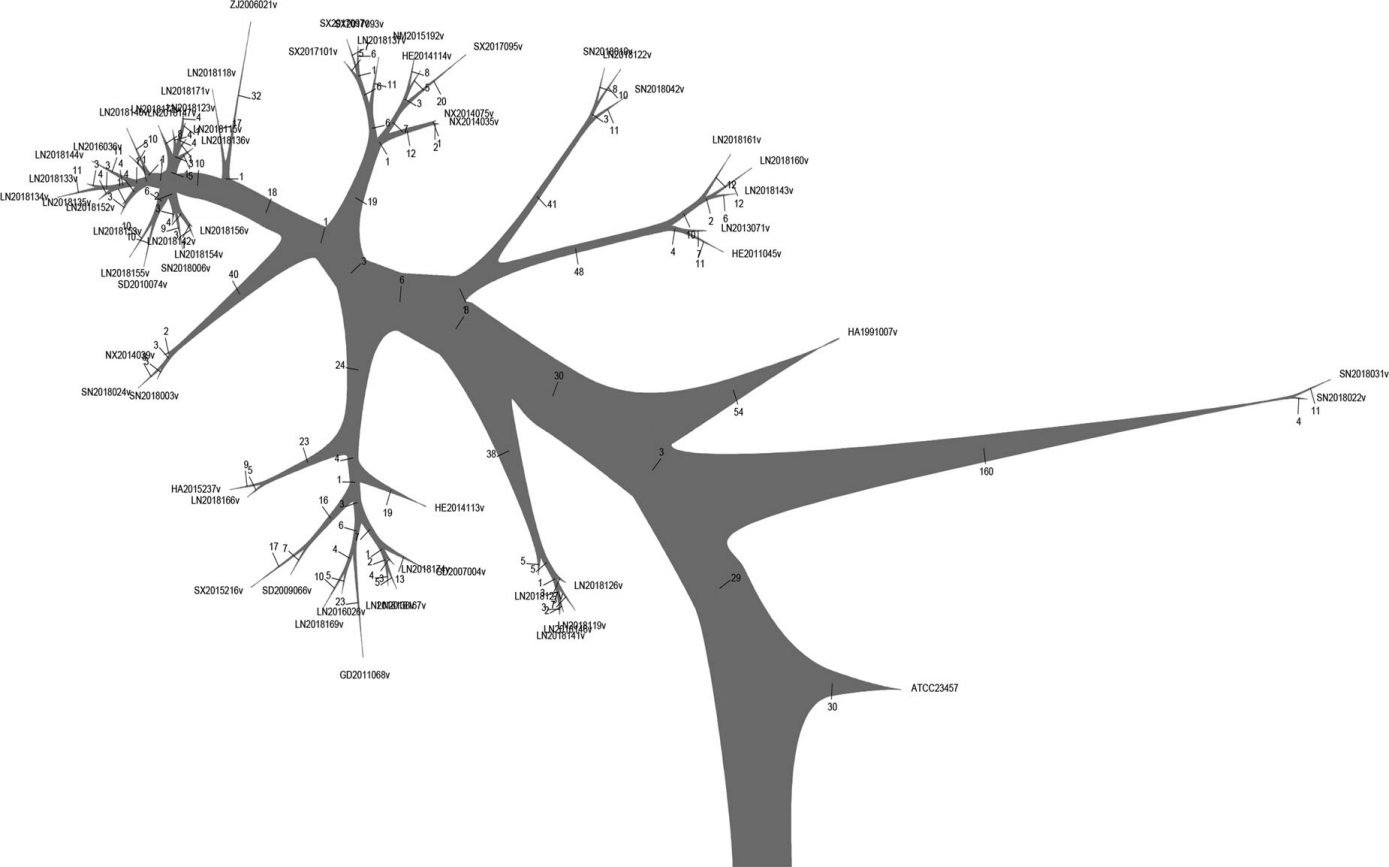
Phylogenetic tree of the 62 rough *B. melitensis* strains by maximal parsimony methods. The root was *B. melitensis* bv.2 str ATCC 23457.

This study constructed a phylogenetic tree of rough *B. melitensis* strains with maximal parsimony (MP) methods and *B. melitensis* bv.2 str ATCC23457 as the root (Figure 4). The results showed that the strain named SN2018022v was the highest similarity with the root strain while the strain named LN2018134v has the greatest difference with the root. Compared with MS tree, the rough *B. melitensis* strains isolated in Liaoning (belonging to complex 1) to be most distant from the root. Its means that the rough *B. melitensis* belonged to complex 1 in Liaoning had the greatest variation from the root.

### 3.3. Metabolic function genes varied in the earlier stage compared to the cellular process and signalling function genes

Compared with known LPS-related genes, SNPs in polysaccharide biosynthesis protein (BMEA_RS02485) were present in all 62 rough *B. melitensis* strains, causing missense variation.

**Figure 5.**
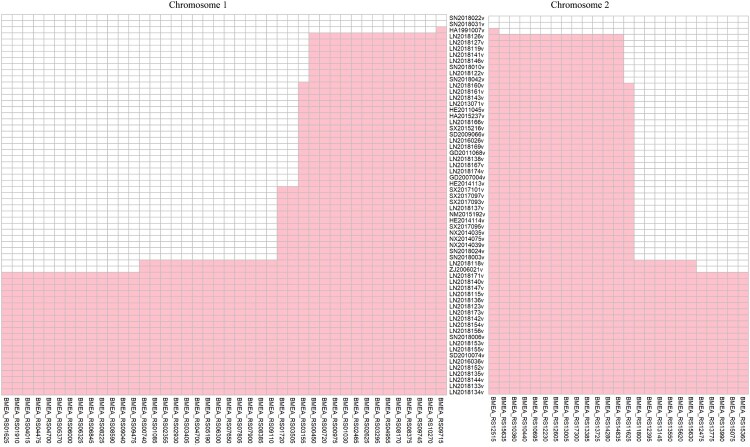
A total of 133 SNPs gradually appeared in the rough *B. melitensis* strains. The left panel shows the SNPs on chromosome 1, and the right panel shows the SNPs on chromosome 2. The middle panel includes the name of the rough *B. melitensis* strains. Each column represents a missense variation gene. The order of the rough *Brucella* strains was arranged according to the MP tree. The closer it was to the middle, the earlier the gene had variations.

The whole genome SNPs of rough *B. melitensis* were analyzed, and the SNPs in all 62 strains and strain-specific SNPs were removed. The order of the rough *Brucella* strains was arranged according to the MP tree. Combined with the MP tree, 133 SNPs (80 on chromosome 1 and 53 on chromosome 2) gradually appeared in the rough *B. melitensis* strains along with the branches. According to the results, the conversions mainly involved A/G (30) and C/T (36), and the amino acid variations mainly involved Lys/Glu (8), Ala/Thr (5), Ala/Val (4), Asp/Gly (3) and Ser/Gly (3); 66 genes carry missense variations (Figure 5).

In addition, functional classification of genes with missense variations was performed by COG analysis. Using BLASTALL, we identified 47 COGs numbers for the 66 genes; 21 clusters had no COGs number, and in those cases, we used “-” instead. In terms of functional assignments, 42% of the gene observed are related to the cellular process and signalling function. Functional genes are enriched in cell wall/membrane/envelope biogenesis (COG category M), transcription (COG category K), and carbohydrate transport and metabolism (COG category G). Combined with the MP tree, metabolic function genes varied in earlier stages than did cellular process and signalling function genes. Some transcriptional regulatory factors (such as the response regulator transcription factor) also varied in later stages. Analysis of the function of genes with missense variations revealed 11 transporter genes. Interestingly, compared to the MFS transporter, the ABC transporter varied in the earlier stage.

### 3.4. No significant difference in rifampicin, doxycycline and streptomycin susceptibility between smooth and rough *B. melitensis* strains

**Figure 6.**
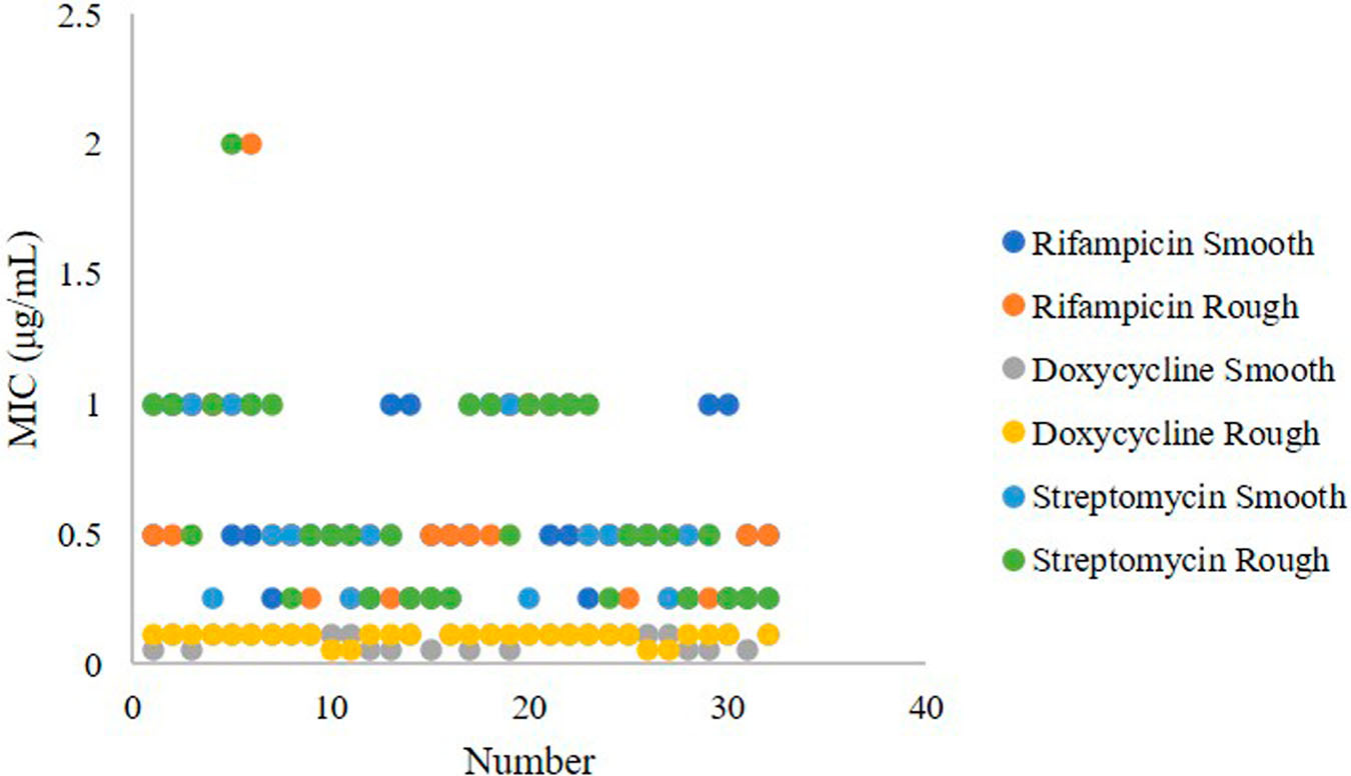
Rifampicin, doxycycline and streptomycin susceptibility in the smooth and rough *B. melitensis* strains. Blue indicates the rifampicin MIC values of smooth *B. melitensis* strains, orange indicates the rifampicin MIC values of rough *B. melitensis* strains, gray indicates the doxycycline MIC values of smooth *B. melitensis* strains, yellow indicates the doxycycline MIC values of rough *B. melitensis* strains, dark blue indicates the streptomycin MIC values of smooth *B. melitensis* strains, and green indicates the streptomycin MIC values of rough *B. melitensis* strains.

Based on the MS trees, 32 smooth and rough *B. melitensis* strains representing each complex were selected for rifampicin, doxycycline and streptomycin susceptibility detection. The rifampin MIC values of the rough *B. melitensis* strains were between 0.25 and 2 μg/mL (only 1 strain had an MIC value of 2 μg/mL), the doxycycline MIC values were between 0.06 and 0.25 μg/mL (only 1 strain had an MIC value of 0.25 μg/mL), and the streptomycin MIC values were between 0.25 and 2 μg/mL, (only 1 strain had an MIC value of 2 μg/mL). The rifampin, doxycycline and streptomycin MIC values of the smooth *B. melitensis* strains were between 0.25–1 μg/mL, 0.06–0.12 μg/mL and 0.25–1 μg/mL, respectively (Figure 6). There was no significant difference in the rifampicin (*p*=0.358), doxycycline (*p*=0.320) and streptomycin (*p*=0.183) susceptibility between the smooth and rough *B. melitensis* strains.

## 4. Discussion

*Brucella* spp. can survive in macrophages by resisting the effects of neutrophils during phagocytosis. As facultative intracellular pathogens, antibacterial drugs and antibodies do not easily enter the intracellular space, leading to chronic brucellosis. Unlike other pathogens, *Brucella* spp. do not have typical virulence factors [24]. The factors affecting *Brucella* virulence are molecular determinants that could help it invade host cells, resist intracellular responses, and survive in professional and non-professional phagocytes [25]. Currently, known *Brucella* virulence factors include LPS, the type IV secretion system (T4SS), the BvrR/BvrS regulation system, outer membrane proteins, and superoxide dismutase (SOD). The LPS of rough *Brucella* spp. lacks the O-antigen. Compared with smooth *Brucella* spp., rough *Brucella* spp. are more easily removed by the host. Importantly, the antibodies induced by rough *Brucella* spp. are different from those induced by smooth *Brucella* spp. Therefore, a smooth varying rough *Brucella* spp. had great advantages in the prevention and control of brucellosis, as the antibodies induced did not affect the serological diagnosis. Moreover, the varying rough *Brucella* spp. conferred protection against *Brucella* re-infection in vivo and no longer displayed residual virulence. The rough variant of the smooth *Brucella* spp. strain retained some of the characteristics of smooth *Brucella* and had a long residence time in mice. Additionally, the varying strain could induce the host to produce humoral and cellular immunity without causing obvious histological damage [26,27]. For this reason, the rough vaccine strain RB51 replaced the traditional smooth strain S19 in several parts of the world [28]. Since December 31, 2000, the United States announced that no cattle had brucellosis, and the vaccine strain RB51 played an important role in the prevention and control of the disease in the United States. However, although strain RB51 has an excellent record in the prevention of brucellosis, its resistance to rifampicin is still a challenge. The rough *B. melitensis* strains analyzed in this study were all spontaneous strains that exhibited almost had no rifampicin resistance. This study also found that the rough phenotype of these strains was stable after several passages (data not shown). Further research will be performed on these strains for screening candidate vaccine strains.

Whole genomic SNPs can be used to trace strains from different locations and periods. One study used the SNPs of whole genomes to construct a phylogenetic tree for evaluating the spread and source of *B. melitensis* around the world and found that *B. melitensis* originated from the Mediterranean [29]. This study also suggested that *B. melitensis* may have spread worldwide by sea through the trade of meat and dairy products [29]. A phylogenetic tree constructed based on SNPs of *B. melitensis* revealed five genotypes, with strains isolated in Asia belong to genotype II [30]. Because fewer Chinese isolates have been published, several strains from different locations and periods in China were sequenced in this study, and the results of the NJ tree were similar to those of previous studies. Although the strains in the complexes 4 and 5 are related to isolates from the Middle East and Russia, most of the isolates from China display some specificity. The rough *B. melitensis* strains mainly clustered in complexes with low similarity to foreign strains. In the NJ tree, the rough and smooth *B. melitensis* strains grouped into the same clades, with no specificity. Therefore, the MS tree was constructed. Several studies have used MS trees to analyze the origin of brucellosis outbreaks. For example, the MS tree constructed using MLVA data in a study in Italy showed that three out of six unknown human cases could be linked to a definite animal source [31].

The phenotypic change from smooth to rough of *Brucella* was widely known. Nevertheless, the smooth to rough phenotypic change was far from being a behaviour exclusively represented by *Brucella*. Several studies had reported on this phenomenon in major *Enterobacteriaceae* [32] species and even in *Mycobacteriaceae* [33]. In *Brucella*, this phenomenon had been reported and was of great importance in basic and vaccine researches [34]. The phenotypic change of *Brucella* under natural conditions mainly existed in two ways: inserting fragments into genes and deletion of genes related to LPS synthesis [16]. In addition, studies had shown that changes in the expression levels of LPS-related genes also caused changes in the phenotype of *Brucella* [35]. This study found that some smooth and rough *B. melitensis* strains isolated in China grouped into the same clades based on the NJ tree. The results of this study were consistent with the facts. And this study also found that the strain with the highest relationship may be a smooth or rough *B. melitensis* strain. The rough *B. melitensis* strains isolated mainly from Liaoning, Shanxi and Shaanxi (Figure 3). The distribution of the rough *B. melitensis* strains was limited and had not caused large-scale spread. Compared with the MS tree distance between Figure 2B and Figure 3, this study found that the MS tree of smooth and rough *B. melitensis* was smaller than the MS tree of rough *B. melitensis* strains. Therefore, the rough *B. melitensis* strains were more likely to variated from smooth strains than the rough strains spread. The rough *B. melitensis* strains in Liaoning Province were mainly isolated from Jingzhou, Huludao and Chaoyang. Based on the MS tree, this study found that the possible sources of the rough *B. melitensis* strains isolated in Liaoning were variated from the smooth *B. melitensis* strains isolated from Liaoning and Inner Mongolia.

Due to the high similarity of isolates from China, the MP tree was chosen when analyzing rough *B. melitensis* strains. Although this study ignored the effect of isolated time during the analysis, it was based on studying *Brucella* from various aspects in different locations. For example, studies of pathogenic isolation and identification have not been performed in some areas of China in the twentieth century. Based on the MP tree, 133 SNPs gradually appeared among rough *B. melitensis* strains along with clades. This study found that the ABC transporter genes varied in earlier stages than MFS transporter genes. The LPS of *Brucella* consists of lipid A, core oligosaccharide, and the O-antigen. Lipid A was synthesized on the cytoplasmic side of the inner membrane by related enzymes; sugars are then linked to lipid A under the continuous action of various glycosyltransferases, followed by transport to the periplasmic side. The O-antigen is synthesized on the cytoplasmic side of the inner membrane and carried to the periplasmic space through the ABC transport system. Finally, LPS is transported to the outside of the outer membrane [36]. In this study, the LPS-assembly protein (BMEA_RS03295) varied relatively early, but the glycosyltransferase (BMEA_RS04475 and BMEA_RS04700) varied relatively late, consistent with the LPS synthesis pathway.

The WHO recommended a treatment for brucellosis in 1989 that involves a combination of doxycycline and rifampicin for six weeks or doxycycline alone for six weeks, followed by doxycycline in combination with streptomycin for 2–3 weeks. This treatment is still recommended for brucellosis. The known rifampicin resistance-related gene in *Brucella* spp. is *rpoB*, and some results showed that variation in the *rpoB* gene caused reduced sensitivity to rifampicin [37]. The latest study showed that some *Brucella* isolates with no variations in *rpoB* are resistant to rifampicin [38,39]. In our study, no variations in *rpoB* of any rough *B. melitensis* strain were observed. Based on the CLSI breakpoints for slow-growing bacteria (CLSI M100-S24), *Brucella* isolates are resistant to rifampin when the MIC value was ≥2 μg/mL. In this study, only one rough *B. melitensis* strain was resistant to rifampicin, MIC = 2 μg/mL. The rifampicin resistance of this strain needs further study. No doxycycline- or streptomycin-resistant strains were found in this study.

## 5. Conclusions

In conclusion, this study used rough phenotype detection and whole genome sequencing methods to analyze the genetic features of rough *B. melitensis* among 199 isolates from China. Drug susceptibility testing was also performed. The results showed that the rough *B. melitensis* strains originated from strains isolated in China rather than foreign strains; based on the MS tree, the strains are related to smooth *B. melitensis* isolates. The NJ tree showed that 9 complexes to be epidemic in China, with rough *B. melitensis* strains were mainly found in complexes 1 and 3. The strains in complex 4 were similar to the isolates from Russia and Turkey (minor), and the strains in complex 5 are more closely related to strains isolated in the Middle East. Moreover, a high association among rough *B. melitensis* strains in Liaoning Province was found, and it is supposed that these strains will spread. For rough *B. melitensis* strains, the metabolic function genes varied in the earlier stages compared to the cellular process and signalling function genes. Some transcriptional regulatory factors also varied in the later stages. ABC transporter genes varied in the earlier stages compared to MFS transporter genes. As there was no significant difference in rifampicin, doxycycline and streptomycin susceptibility between the smooth and rough *B. melitensis* strains, the treatment of brucellosis is not affected by strain phenotype. This study provides important findings for understanding the genetics and evolution of rough *B. melitensis* in China.

## Conflict of interest

Neither of the authors of this paper has a financial relationship with other organization that could inappropriately influence or bias the content of the paper.

## Supplementary Material

Table_S2.xlsx

TableS1.xlsx
